# A Rare Case of Diffuse Large B-Cell Lymphoma Found in the Subcutaneous Scalp

**DOI:** 10.7759/cureus.35245

**Published:** 2023-02-21

**Authors:** Kristin N Slater, Moustapha Doulaye, Adnan Mohammadbhoy

**Affiliations:** 1 General Surgery, Lincoln Memorial University-DeBusk College of Osteopathic Medicine, Harrogate, USA; 2 General Surgery, Bravera Health Seven Rivers, Crystal River, USA

**Keywords:** subcutaneous malignancy, scalp malignancy, subcutaneous tumor, subcutaneous scalp tumor, scalp tumor, diffuse large b-cell lymphoma of the scalp, extra-nodal, extra-nodal involvement, diffuse large b-cell lymphoma, extranodal diffuse large b-cell lymphoma

## Abstract

Subcutaneous scalp manifestations of diffuse large b-cell lymphoma are uncommon and can be an easily overlooked diagnosis. Today we report a rare case of a 60-year-old male with a previous history of treatment and removal of multiple benign cysts who presented to the office for the removal of a subcutaneous mass on the left occipital scalp. Intraoperatively the mass did not resemble a cyst. Pathology results showed diffuse large b-cell lymphoma, highlighting the importance of thorough management of subcutaneous masses.

## Introduction

Diffuse large b-cell lymphoma (DLBCL) represents approximately one-third of all non-Hodgkin’s lymphomas [1]; extranodal presentation on the scalp is rare [2-3]. Rare extranodal presentations documented in the literature include the scalp, testicles, bone, bladder, and nasal septum, whereas, the gastrointestinal tract is the most common extranodal site [2-9]. Skin involvement in DLBCL can occur as a primary or secondary manifestation, which can be difficult to differentiate [1, 4]. Different subclassifications can have different prognoses and require different treatments [1]. In these rare cases, the primary extranodal masses can present as subcutaneous or cutaneous involvement mimicking a subcutaneous cyst [1-4]. Primary cutaneous large b-cell lymphoma is a subclass that presents without evidence of internal or disseminated disease [1]. The likely involvement of cervical lymph nodes combined with the immunophenotype suggested that our case was DLBCL with extranodal presentation, which was later confirmed by a positron emission tomography (PET) scan. After excisional removal of the extranodal site, the patient followed up with oncology for further evaluation and treatment.

## Case presentation

A 60-year-old white male presented to the clinic for the removal of a subcutaneous mass on the left posterior scalp that had been present for 1-2 months. The patient’s medical history was significant for sebaceous and pilar cysts, hypogonadism, chronic kidney disease, hyperlipidemia, hypertension, depression, and anxiety. The patient’s surgical history included shoulder surgery and cyst excisions. His previous cyst excisions were a combination of sebaceous and pilar cysts that had been located on the back, face, and scalp, but none had been located on the left posterior scalp where this lesion presented. The mass was approximately 5cm x 5cm in size and presented as a smooth solitary subcutaneous mass without skin involvement or ulceration. No associated cellulitis or drainage. It was smooth and oval, longer horizontally and located midline along the left lower posterior scalp. The patient had noted pain with pressure or palpation to the area and difficulty lying on the left side of the head. Left unilateral anterior cervical lymphadenopathy was appreciated on exam. The patient was treated on the clinical diagnosis of an infected sebaceous cyst and was previously prescribed a 10-day course of doxycycline without improvement.

Excision by general surgery was performed. Intraoperatively the mass did not appear to resemble a cyst, nor did it have a cystic consistency. The mass was tan-white, rubbery, and fixed to the surrounding tissues during the removal. Successful removal showed an ellipse of skin and underlying soft tissue measuring 4.5 x 2.1 x 1cm. The skin surface showed marked irregularity. Sectioning revealed diffuse tan-white induration of the subcutaneous soft tissues.

**Figure 1:**
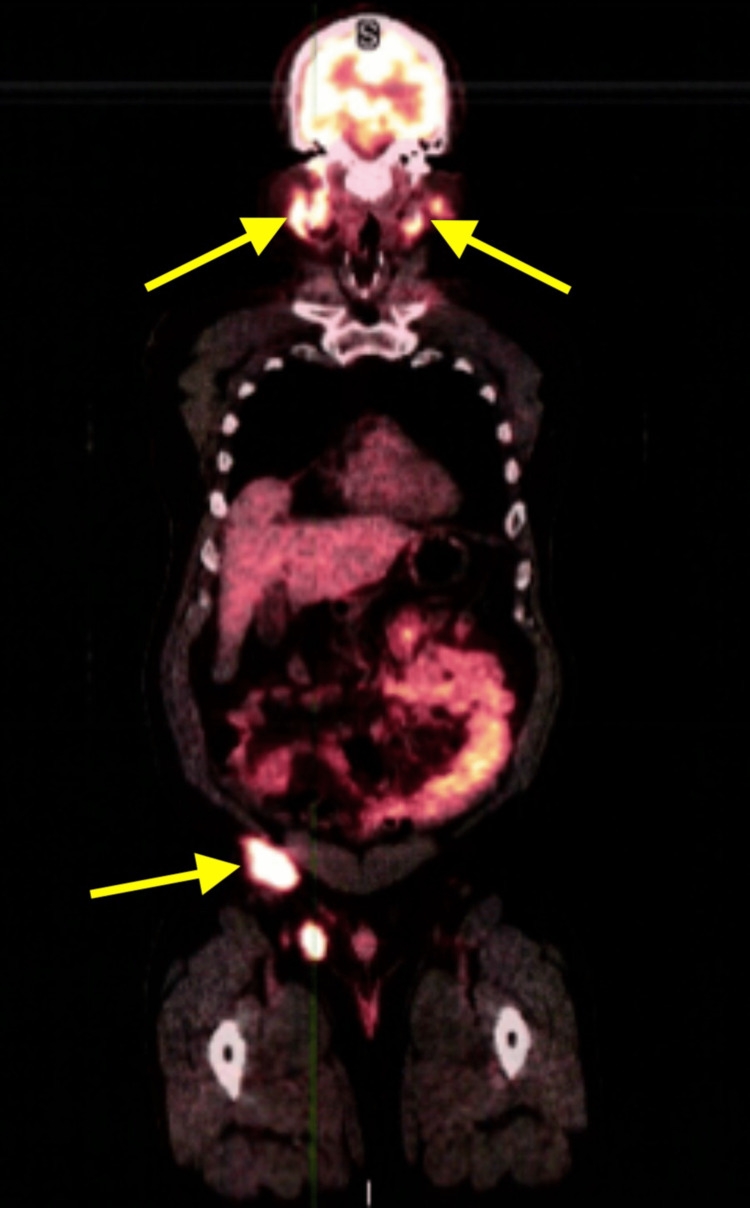
PET scan showing diffuse lymphoma involving both above and below the diaphragm

**Figure 2:**
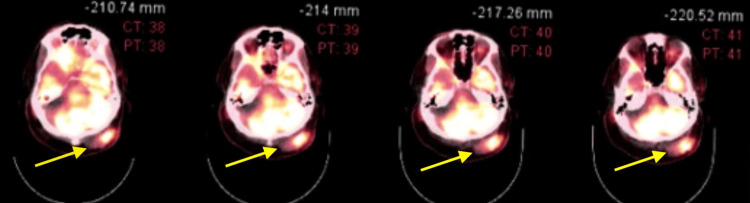
PET scan showing subcutaneous lymphoma involving the scalp posterior to the left occipital bone

Pathology results showed a diffuse infiltrate of lymphoid cells with a bimodal pattern of large and small cells showing mitotic activity and scattered foci of necrosis seen throughout, consistent with a malignant lymphoma. Further special staining revealed tumor cells to be positive for CD20, CD10, and BCL2. There was focal weak positivity for BCL6. The tumor cells were negative for CD3, which stains the background reactive T cells. The tumor is negative for CD5, C-MYC and MUM1. Immunostains for kappa and lambda light chains were non-contributory. High proliferative activity (80%) is seen on immunostain for KI-67. The histologic appearance and immunophenotype fit best with the diagnosis of diffuse large b-cell lymphoma. A positron emission tomography (PET) scan was ordered showing diffuse lymphoma involved both above and below the diaphragm (Figure 1) and subcutaneous lymphoma involving the scalp posterior to the left occipital bone (Figure 2). The patient was referred to oncology for treatment and management.

## Discussion

This case describes the rare finding of an extranodal subcutaneous manifestation of DLBCL. The patient’s history of multiple benign cysts of various sizes could have made the diagnosis easy to miss, assuming a presumptive diagnosis of a benign cyst. The presentation described today is not often seen [2-3] and is considered a high-grade lymphoma [4-5]. Of all forms of DLBCL, extranodal forms only comprise 34.48% [10]. The gastrointestinal tract has been noted to be the most common extranodal site [8]. Although, any extranodal site could be the site of primary involvement [8].

Rare sites of extranodal DLBCL have been documented in the literature, including presentations in the scalp, testicles, bone, bladder, and nasal septum [2-3, 6-9]. A recent 2022 study found that the percentages of extranodal DLBCL from years 2000-2015 at the age of 18 or greater in their search were as follows: GI tract 28.31%, nervous system 13.78%, head and neck 11.79%, skin and soft tissue 9.54%, genitourinary 7.24%, musculoskeletal 5.80%, respiratory tract 5.17%, pancreas and hepatobiliary 4.16%, endocrine organs 3.66%, heart and mediastinum 3.28%, breast 2.49%, bone marrow 2.47%, and others 2.30% [10]. Extranodal DLBCL was found to be more common in non-Hispanic white males 60 years of age or older [10]. Our patient falls within these demographic parameters as a 60-year-old, white male. The cause of DLBCL is multifactorial with potential risk factors including, but not limited to, genetic susceptibility, environment, and immune dysregulation [5].

Standard management of DLBCL in advanced disease includes six cycles of rituximab, cyclophosphamide, doxorubicin, vincristine, and prednisone (R-CHOP therapy) every three weeks, which can be curative in 60% of cases, but DLBCL resistant to R-CHOP therapy has unfavorable outcomes [5]. DLBCL with MYC and BCL2 and/or BCL6 rearrangements are noted to have particularly unfavorable outcomes after R-CHOP therapy [5]. Our patient fortunately was negative for MYC, showing positive staining instead for BCL2, and focal weak positivity for BCL6, and is undergoing treatment with R-CHOP therapy. The majority of DLBCL cases are detected at a late stage [5], which was the case in our patient as his PET scan showed lymphoma both above and below the diaphragm. The outcomes can vary based off the subtype of DLBCL, and its corresponding response to conventional therapies [5].

Our hope is that our case can offer a presentation of malignancy that can be detected during routine examinations. This case acts as a reminder to remain diligent in screening for and treatment of subcutaneous masses even in cases where the patient has a history of multiple benign subcutaneous growths.

## Conclusions

Subcutaneous presentations of DLBCL are a rare finding. When managing scalp masses, a diagnosis of DLBCL should be considered and a thorough workup should be completed. A history of benign subcutaneous lesions does not mean rarer conditions such as DLBCL should be excluded from the differential. This case highlights the importance of a thorough workup of subcutaneous skin lesions and offers a unique and rare presentation of DLBCL to monitor for.

## References

[1] Khatib Y, Dande M, Patel RD, Makhija M (2017). Primary cutaneous large B-cell lymphoma of scalp: case report of a rare variant. Indian J Pathol Microbiol.

[2] Nasirmohtaram S, Mesbah A, Mazloom F (2022). An extremely rare presentation of non-Hodgkin lymphoma in the head and neck: a case report. Egypt J Otolaryngol.

[3] Hasturk AE, Eyupoglu EE, Gel G, Gokce C (2019). P14.14 Scalp invasion of diffuse large b-cell lymphoma without systemic involvement. Neuro Oncol.

[4] Kilaru S, Panda SS, Mishra S (2021). Cutaneous involvement in diffuse large B cell lymphoma at presentation: report of two rare cases and literature review. J Egypt Natl Canc Inst.

[5] Sehn LH, Salles G (2021). Diffuse large B-cell lymphoma. N Engl J Med.

[6] Trama F, Illiano E, Aveta A, Pandolfo SD, Bertuzzi G, Costantini E (2021). Bilateral primary testicular diffuse large B-CELL lymphoma. Urol Case Rep.

[7] Ayesh Haj Yousef MH, Audat Z, Al-Shorafat DM, Al-Khatib S, Daoud AK (2022). Primary diffuse large B cell lymphoma of bone: a single-center experience. J Blood Med.

[8] Zanelli M, Sanguedolce F, Zizzo M (2022). Primary diffuse large B-cell lymphoma of the urinary bladder: update on a rare disease and potential diagnostic pitfalls. Curr Oncol.

[9] Patel V, Tacy CJ, Creamean T, Sibia A, Patel J (2022). Atypical location of diffuse large B-cell lymphoma in the nasal septum. Cureus.

[10] Gupta V, Singh V, Bajwa R (2022). Site-specific survival of extra nodal diffuse large B-cell lymphoma and comparison with gastrointestinal diffuse large B-cell lymphoma. J Hematol.

